# Editorial: Neuroinflammatory and oxidative/nitrosative pathways in neuropsychiatric and neurological diseases and their possible neuropharmacological regulation, Volume II

**DOI:** 10.3389/fphar.2023.1191907

**Published:** 2023-03-31

**Authors:** Marta P. Pereira, Borja García-Bueno, Javier R. Caso

**Affiliations:** ^1^ Departamento de Biología Molecular, Centro de Biología Molecular “Severo Ochoa” (UAM-CSIC), Madrid, Spain; ^2^ Instituto Universitario de Biología Molecular, IUBM, Universidad Autónoma de Madrid, Madrid, Spain; ^3^ Departamento de Farmacología y Toxicología, Facultad de Medicina, Universidad Complutense de Madrid (UCM), Instituto de Investigación Sanitaria Hospital 12 de Octubre (Imas12), Instituto Universitario de Investigación Neuroquímica (IUIN-UCM), Madrid, Spain; ^4^ Centro de Investigación Biomédica en Red de Salud Mental, Instituto de Salud Carlos III (CIBERSAM, ISCIII), Madrid, Spain

**Keywords:** neuropsychiatric diseases, neurological diseases, neuroinflammation, oxidative/nitrosative stress, neuropharmacology

The rationale for this Research Topic still is the same as the reasoning described in the Volume I (Pereira et al., 2022). Briefly, despite the upswing of neurological and neuropsychiatric diseases as prominent sources of disease burden, incapacity, and mortality (Murray et al., 2012) there are important questions about their etiology without an answer yet, and for that reason, there is vital need to characterize novel therapeutic targets and pathophysiological paths involving these disorders. Hence, this Volume II offers new studies and theoretical approaches from the standpoint of the neuropharmacology, always taking into consideration the important body of knowledge indicating that the immune activation and the ensuing oxidative/nitrosative harm are implicated in these illnesses (Caldwell et al., 2020).

Zhou et al. revised the latest advances in the realm of microglia-mediated hypoxic-ischemic inflammation, specifically reviewing the works regarding the perinatal hypoxia-ischemia, the most frequent foundation of acute neonatal brain injury. Furthermore, they focused on how histone lactylation can be a regulator of inflammation by influencing macrophage activation. According to their review, it appears like the epigenetic reprogramming-associated lactate input is connected to disease aftermaths. For that reason, authors conclude that expanding the understanding of the shared interactions concerning histone lactylation and inflammation could guide to the development of novel immunomodulatory therapies for brain damage in newborns, which is a field warranting further consideration.

Evans et al. reviewed what is acknowledged about the mechanisms causing the vulnerability of noradrenergic neurons of the Locus coeruleus to degeneration both with normal aging and in neurodegenerative disorders, considering the role of noradrenaline as dual modulator of cognition and neuroinflammation. Moreover, this article also reviews therapeutic strategies for protecting Locus coeruleus noradrenergic neurons from oxidative stress and neurodegeneration. Authors conclude that future endeavors should be focused on the exposition of mechanistic paths prompted by risk factors (i.e., systemic inflammation, sleep disruption, chronic stress, and viral infections) guiding to selective neuronal cell loss in key brain regions, such as the Locus coeruleus To identify brain areas in which the neuronal cell loss occurs is crucial, and this review helps to understand its importance.

Obuchowicz et al. analyze, employing rats, the consequences of prenatal administration of imipramine and venlafaxine (a tricyclic antidepressant and a serotonin-norepinephrine reuptake inhibitor, respectively) on morphology and activity of a primary glial culture. The prenatal administration, employing an oral gavage, was considered an inductor of prenatal stress, causing shifts in the glial cultures, such as a higher percentage of microglia in the mixed glial cultures and a higher percentage of dead cells. Results from this study seems to indicate that the exposure to mild forms of prenatal stress might predispose subjects to neurodevelopmental and psychiatric disorders and that venlafaxine could be a good therapeutic approach, at least judging by its effects on microglia, which is a significant supervisor of the development of the central nervous system. To test current approved drugs is always an interesting approach. Therefore, to identify one approved antidepressant as a potential treatment for actions of the prenatal stress on microglia, as it has been done in this research, is relevant, although further research is very much needed.

Jaffa et al. assessed the means through which plasma prekallikrein participates in microglial activation and the production of proinflammatory cytokines through the study of the prekallikrein-related receptors and their dynamics. This study has an important innovative component, as plasma prekallikrein has been recently described as a parameter involved in neuroinflammatory processes, and as a result, its role in the activation of microglia is relatively unknown. Employing murine microglial cells, bioinformatics analysis, and different pharmacological tools, authors were able to show that prekallikrein can modulate microglial activation through the recruitment of the bradykinin 2 receptor and the protease-activated receptor 2, and acting as a regulator of neuroinflammatory responses which could have a potential use in the future in the prevention of the development of neurodegenerative diseases.

Yu et al. gathered significant articles in the Web of Science Core Research Topic database from 2010 to 2022 and analyzed them, aiming to grasp the most important numbers about the bibliography concerning the role of the inflammasome NLRP3 in the realm of neurological diseases. After retrieving a total of 1,217 research articles and reviews, authors concluded that the research about NLRP3 and neurological diseases is in good shape and thriving. Data indicate that Chinese scholars contributed the most significant number of articles, while researchers from developed countries presented more influential papers. But more importantly, data show that although inflammasomes are rising as potential therapeutic targets for the neurological disorders treatment, there is still an important need for deeper research about the role of the NLRP3 inflammasome in diseases of the central nervous system.

Finally Vera-Montecinos et al. identified a central role of RAB7A in a modified molecular network implicated in immune response in the *postmortem* dorsolateral prefrontal cortex (DLPFC) from subjects with schizophrenia. It has been theorized that the transformed cognitive functioning in schizophrenia would be caused by the dysfunction in the maturation of connectivity between different brain areas and the prefrontal cortex. Thus, the results obtained in this article could offer a series of proteins related to dysfunction in the immune response or involved in negative symptoms in schizophrenia, increasing the knowledge about potential targets for new pharmacological strategies for amending cognitive and negative symptoms.

To conclude, this Research Topic strengthens, once more, the significance of the immune system responses, particularly neuroinflammation, and oxidative stress processes as crucial participants in the etiology of neuropsychiatric and neurological disorders. Above all, in this Research Topic it has been pointed out the importance of studies covering several areas of research through different means, such as the study of epigenetic reprogramming, the recognition of specific brain areas involved in the pathophysiology of these diseases, the research about the actions of known drugs in different animal models, the search for new biomarkers, and the pursuit of new potential targets for the treatment of different facets of the schizophrenia, such as the negative and cognitive symptoms.
